# The Contribution of Dietary Magnesium in Farm Animals and Human Nutrition

**DOI:** 10.3390/nu13020509

**Published:** 2021-02-04

**Authors:** Luciano Pinotti, Michele Manoni, Luca Ferrari, Marco Tretola, Roberta Cazzola, Ian Givens

**Affiliations:** 1 Department of Health, Animal Science and Food Safety (VESPA), Università di Milano, 20133 Milan, Italy; michele.manoni@unimi.it (M.M.); luca.ferrari2@unimi.it (L.F.); marco.tretola@agroscope.admin.ch (M.T.); 2 CRC I-WE (Coordinating Research Centre: Innovation for Well-Being and Environment), Università di Milano, 20133 Milan, Italy; 3 Agroscope, Institute for Livestock Sciences, 1725 Posieux, Switzerland; 4 Department of Biomedical and Clinical Sciences “L. Sacco”, Università di Milano, 20157 Milan, Italy; roberta.cazzola@unimi.it; 5 Institute for Food, Nutrition and Health, University of Reading, Reading RG6 6AR, UK; d.i.givens@reading.ac.uk

**Keywords:** magnesium supplementation, animal nutrition, livestock, magnesium deficiency, magnesium in human nutrition, animal-derived foods

## Abstract

Magnesium (Mg) is a mineral that plays an essential role as cofactor of more than 300 enzymes. Mg in farm animals’ and human nutrition is recommended to avoid Mg deficiency, ensure adequate growth and health maintenance. Mg supplementation above the estimated minimum requirements is the best practice to improve farm animals’ performances (fertility and yield) and food products’ quality, since the performance of farm animals has grown in recent decades. Mg supplementation in pigs increases meat quality and sows’ fertility; in poultry, it helps to avoid deficiency-related health conditions and to improve meat quality and egg production by laying hens; in dairy cows, it serves to avoid grass tetany and milk fever, two conditions related to hypomagnesaemia, and to support their growth. Thus, Mg supplementation increases food products’ quality and prevents Mg deficiency in farm animals, ensuring an adequate Mg content in animal-source food. These latter are excellent Mg sources in human diets. Sub-optimal Mg intake by humans has several implications in bone development, muscle function, and health maintenance. This review summarizes the main knowledge about Mg in farm animals and in human nutrition.

## 1. Introduction

The average content of Mg in the body of most animals is ~0.4 g Mg per kilogram of body weight [1]. In the human body, the total Mg concentration is around ~20 mmol/kg of fat-free tissue. This value corresponds to ~24 g of total Mg in an average 70 kg adult with 20% (*w*/*w*) fat [2,3]. In comparison, the body content of calcium is ~1000 g (i.e., 42 times greater than the body content of Mg) [4]. Assuming that a similar relationship exists for other mammals, the total body Mg^2+^ of a cow with a body weight of 700 kg should be roughly 455 g, of which approximately 320 g would be skeletal (approximately 60–70% of Mg is located in the skeleton), about 130 g intracellular, while only about 4–5 g would be found in the total extra-cellular space (i.e., 35% is distributed in soft tissue and extracellular fluid) [4,5]. For the same cow the calcium content is between 7–9.6 kg, which means ~21 times greater than the body content of Mg. 

However, Mg is important for many functions in animals’ body and its deficiency results in several dysfunctions. Accordingly, as reported for humans, also in the case of farm animals Mg requirements and recommendations have been defined. 

In light of this, the aims of the present review are to: (i) provide an overview of Mg requirements and recommendations in farm animals; (ii) describe the main effects of Mg supplementation on growth, reproduction, health and product quality in farm animals; (iii) describe the potential contribution of food of animal origin to the Mg intake in humans; (iv) discuss the consequences on humans’ health of sub-optimal Mg intake, which are rather different to those in farm animals.

## 2. Mg in Farm Animals’ Diet

Mineral nutrients are essential for adequate growth, productivity, and health of all food producing animals. Among minerals, Mg is considered one of the seven macro minerals that are essentials in farm animal diets. These are: calcium (Ca), phosphorus (P), magnesium (Mg), sulphur (S), sodium (Na), chlorine (Cl), and potassium (K). Many factors can affect mineral requirements of farm animals, namely: species, age, physiological stage, and performance (average daily gain, milk yield, egg yield, etc.). The performance and efficacy—expressed as feed conversion rate (FCR, kg feed per kg of animal product)—of modern high producing farm animals has increased dramatically over the past decades (Table 1), which may contribute to the changes in nutritional requirements of food producing animals. Although the requirement for Mg can be met by common feed ingredients in animal diets, research and practice have shown benefits from supplementing Mg above the estimated minimum requirements in several food producing animals like pigs, poultry, and cows (as farm ruminants’ representative). The practice of supplementing feedstuffs with Mg is widely used, with the primary aim to avoid Mg deficiency and then to improve animal performance (fertility and yield) and sometimes products’ quality [4,5,6,7,8,9,10].

As for other farm animal species, Mg is a key dietary element and it is essential for animal growth and survival. Notably, it has essential functions in cellular metabolism and bone development [2,13]. In terms of supplementation, oxide, carbonate and sulphate are all sources of highly available Mg for farm animals [14]. Generally, Mg oxide (MgO) is the most used and the highest Mg-concentration mineral source available as an animal feed ingredient (Table 2). Magnesium oxide usually guarantees an adequate absorption of Mg ions. Not all sources of MgO are equal to the task of providing efficiently the necessary Mg^2+^ ion amount to a living organism. Solubility, reactivity, and bioavailability are all characteristics that differ from one MgO product to another [4]. The mineral feed bioavailability is also different: for example, the average Mg bioavailability of magnesium oxide, compared to magnesium phosphate, is around 20 vs. 45% [15].

The recommendations of the National Research Council (NRC) for different farm species are as follows: 400 mg/kg Mg dry matter (DM) for pigs [16], 500 mg/kg Mg DM for broilers, turkey poults and laying hens (with a food intake of 100 g/day) [17]. A different scenario exists for ruminant animals (beef and dairy cattle, sheep, and goat). Insufficient absorption or availability of Mg in ruminants leads to Mg deficiency which manifests in clinical signs such as tetany (grass tetany) or parturient paresis (milk fever). Intuitively, excessive Mg supplementation has also some detrimental effects. In farm animals, diarrhea is the most obvious effect of high intake of Mg. Very high dietary Mg intake (e.g., about seven times fold the minimum requirement for pigs) [18] can reduce feed consumption and weight gain.

However, combining quantities of Mg recommended in each species per kg of metabolic weight (body weight^0.75^; Table 3), it is evident that the quantities recommended for pig and poultry are higher than ruminants. These differences might depend from several factors that can be linked to the animals and their diets. Poultry and pigs are omnivorous species, with very fast growth rates that reach in modern breeds 100 g and 1 kg/day, respectively. These figures speak for themselves. Such growth performance needs a lot of energy and nutrients including minerals. Cow, considered as the reference ruminant’s animal in the present work, is an adult herbivorous animal in which the Mg absorption and metabolism, starting from the rumen, is different and in which the main output is in milk. The lowest values reported for cow probably explain its sensitivity to the Mg deficiency especially at the onset of lactation (e.g., milk fever). By contrast, the recommended quantities in humans (see below) are enough to reach an adequate steady-state condition in typical adult male humans (maintenance).

## 3. Mg Supplementation in Pig Nutrition

The minimum Mg requirement for pigs receiving a purified diet is 325 mg/kg DM and, accordingly to NRC [16], 400 mg/kg DM are recommended. Higher supplementations have been reported for optimum growth and reproductive performance in pigs (400–500 mg/kg DM). Thus, the dietary intake of 400 mg/kg is considered sufficient and 500–650 mg/kg Mg is recommended for pigs. On the other hand, the demand for Mg increases proportionally to the protein content of the diet [15]. Deficiency symptoms in pigs include a strong response of the nervous system (hypersensitivity, anxiety, fear), muscle contractions and a drop in productivity (a slower growth rate because of loss of appetite). The kidney is the major site of Mg homeostasis and is able to excrete Mg at high dietary concentrations and reabsorb Mg with greater efficiency at low dietary concentrations. 

In terms of sources, Mg can be found in several feeds, such as green forage, animal derived feed, and mineral supplements. Feed ingredients like wheat bran, dried yeast, linseed meal, and cottonseed meal are good sources of Mg. The average content (g/kg DM) of Mg in cereals, oil meals and fish meals is: 1.1–1.3 g, 3.0–5.8 g, and 1.7–2.5 g, respectively [15]. However, when Mg digestibility is considered, these figures must be reconsidered: in common pig feeds only 20 to 30% is digestible [18]. For this reason, supplements like MgO are commonly used in pig formulas. As in the other non-ruminant animals (pigs and poultry), Mg is absorbed primarily in the small intestine, at an efficiency of approximately 60%, mostly via passive transport. In this site, potassium, calcium and ammonia are its antagonists [15].

### 3.1. The Effects of Mg on Meat Quality 

Regarding pigs, a nutritional regime is one of the key environmental factors affecting fattening results, farm financial return and meat quality. Dietary Mg supplementation in pigs has generally been ineffective for increasing growth of fattening pigs (average daily gain), but has been observed to improve pork quality [18], specifically colour and drip loss [19]. 

Colour is one of the most important meat quality characteristics. It is a visual element that depends on the presence of pigments, the tissue composition, and texture of meat. There is a correlation between meat colour and the pH of muscles. Changes in meat colour are, in 50% of cases, determined by pH values measured 24 h post-harvest. Meat appearance is positively affected by nutritional factors, such as vitamin C, vitamin E, selenium, and Mg content. In post-harvest processes in muscles, glycogen is converted into lactic acid and the pH of meat decreases, leading to the occurrence of Pale Soft Exudative (PSE) meat defects. The PSE is a condition that occurs usually during the conversion of muscle to meat. PSE has been documented mostly in pork carcasses, even though it is also reported in other species. The typical pH in pork would be 6.5–6.7 with a temperature of 37 °C at 45 min post-mortem. However, in unusual carcasses, the pH may drop to 6.0 in the same time period. In this latter case, the combination of rapidly decreasing pH and high carcass temperature results in the denaturation of some of the contractile proteins, with consequent loss of water holding capacity (drip loss). Denatured proteins are not capable of holding or binding muscle water, as well as fully native proteins. More specifically, the length of the myosin filaments decreases by 8–10% during this process. PSE meat is usually of pale colour, wet in appearance, and very soft in texture, thus making PSE one of the major quality defects in meat industry [20]. This defect reduces consumer’s acceptability, shelf life, and yield of meat, thus affecting profits tremendously. To cope with this problem, it has been shown that Mg inhibits stress-induced glycolysis, thus improving meat quality [21,22]. That’s why the addition of Mg to finisher diets has been found to reduce the incidence of PSE meat from 15 to 50% of carcasses. Therefore, adding this mineral could decrease drip loss and improve meat colour from 3.6 to 6.6% with a short-term administration. Specifically, Mg improves colour stability and reduces drip loss. 

Mg supplementation is a relatively easy method of improving pork quality [18]. Animal diets can be supplemented with organic (proteinate, aspartate) or inorganic (oxide, sulphate, chloride, phosphate) forms of Mg. A good solution to obtain this effect is to add Mg to drinking water: the administration of 600 mg of Mg per litre of water, for two days before slaughter (short term), has been found to be also effective [23]. 

### 3.2. Mg for Sows

The reproductive performance of high producing sows has increased dramatically over the past decades, which may contribute to the changes in their nutritional requirements. It has been proven that Mg supplementation improves the conception rate of sows by 11–15% [10]. Moreover, its supplementation significantly reduces the weaning to oestrus interval in gilts and enhances the total number of born piglets, born alive, and weaned. This increase is particularly evident for sows fed with 150–300 mg/kg of supplemental Mg (basal diet contains 210 mg/kg of Mg). The improvement of sows’ performance may be related to a reduced incidence of constipation, which has been shown to negatively affect the reproductive performance of sows. 

In addition, the increased levels of Mg in sows’ lactation diet has a repercussion on its concentration in colostrum, as well as in the serum of piglets. This has been recently reported by Zang et al. [10], who evidenced that the increase in Mg content in sow’s lactation diets can lead to the increase, not only of the concentration of Mg in colostrum, but also of the serum Mg concentration in suckling piglets. These results highlight the role of the maternal diet in defining piglets’ nutritional status (e.g., their Mg status).

However, these effects observed in sows appeared to be age-related, which may be due to depleted body stores of minerals in high producing sows as they age [24]. Therefore, it is possible that, as the sows age, Mg stores in their body decline, increasing the reliance on the diet to provide it. In addition, dietary Mg supplementation positively affects pork quality by enhancing meat colour and reducing drip loss. 

Mg supplementation also improves sows’ fertility (e.g., conception rate) and helps during pregnancy in controlling constipation problems. Furthermore, the increase in dietary Mg in lactating sows leads to the increase in both Mg colostrum content and Mg serum content of suckling piglets (i.e., their Mg status). 

## 4. Mg Supplementation in Poultry Nutrition

The minimum Mg requirement for broilers, turkey poults, and laying hens is around 500 mg/kg DM, accordingly to NRC [17]. Mg supplementation in poultry is affected by the growth rate and reproductive performance [6], but it is usually suggested after the third week of age, for preventing leg bones malformation. After this phase, Mg supplementation is recommended specially to prevent its deficiency. Indeed, Mg deficiency in avian species could lead to serious biochemical and symptomatic variations: for example, in young poultry (older than 3 weeks), it has been observed that it caused poor growth of body and feathering, decreased muscle tone, incoordination, squatting, fine palpable tremors, convulsive attacks, coma, and ultimately death [7]. In laying hens, the symptoms are different: reduced egg production, decreased feed consumption, nervous tremor, and seizures are the most reported deficiency signs. By contrast, adequate Mg supplementation in poultry exerts beneficial effects, increasing weight gain of broilers and meat quality, and egg production of laying hens. The influence of increased Mg levels fed to parent stock on progeny performance is another area of interest. Parent stock’s breeders supplementation with Mg (up to 500 mg/Mg/day) positively affects egg quality and hatchability [4,6]. Recent results also showed that MgO supplementation improved FCR and skeletal integrity [4,7,25] and exerted a positive effect on pullet skeletal development, body weight and onset of egg production [26].

### Interaction with Ca and P

Mg metabolism is closely associated with Ca and P. These are two important minerals for laying hens that affect productive performance and eggshell quality. The use of Ca and P compounds appears to be determined largely by the relative proportions in which these elements and Mg are present in the ration. The commercial diet of chickens younger than 3 weeks of age should not be supplemented with Mg, as this leads to leg bone malformation and development of perosis-like symptoms. An antagonistic relationship also seems to exist between Ca and Mg in relation to skeletal integrity and eggshell quality in laying hens. An increased dietary Mg supply in laying hens, although not affecting Ca retention, reduces eggshell Ca content and bone Ca content, whereas shell Mg content is increased [7]. The variety of mechanisms related to Mg-Ca interaction demonstrates the need of close regulation of any variation in Mg level in poultry diets. Nutritionists today strive for optimisation of P content in poultry diets because of the high costs of P supplements, finiteness of phosphate rock supply and negative ecological impact of high P excretions. A supplementation with extra-nutritional levels of Mg to commercial poultry feed may disturb P as well as Ca availability, and thus negatively impacting bird performance and bone mineralization, especially in laying hens [27]. From another point of view, other dietary constituents can affect Mg bioavailability, retention and finally Mg status of poultry. Among these, the phytate effect is one of the most known: dietary phytate generally decreases Mg absorption in poultry through the formation of insoluble Ca-Mg-phytate complexes under the pH conditions of the small intestine. Use of phytase enzymes (common practice in poultry diets) might prevent this detrimental effect [28].

Mg is an essential element in poultry nutrition. Although most compound feeds for poultry contain Mg to an extent that makes Mg deficiency unlikely under practical conditions, other dietetic features of poultry formulas merit attention. Indeed, in specific poultry compound feeds (e.g., laying hens, breeders, specific Ca and P ratios, presence of phytate, etc.) Mg supplementation can be recommended for designing balanced diets aimed at achieving maximal performance. 

## 5. Mg Supplementation in Cow Nutrition

In dairy and beef cows’ diets, Mg is generally recommended at 1.2 to 3 g/kg DM [29,30]. An adequate dietary supply of Mg supports animal’s health and prevents deficient conditions. The most important deficient conditions are grass tetany and milk fever. Grass tetany is a clinical sign of hypomagnesaemia in cows, in which Mg level in cerebrospinal fluid decreases below a critical level (<0.7 mmol/L), following a decrease in blood plasma. This impairs the synaptic activity of neurons and causes symptoms such as excitement and muscular spasms (tetany). It is recognized that the incidence of grass tetany in cows is related to the fertilization of pastures with fertilizers containing K, which impairs Mg absorption. Milk fever (or parturient paresis) is another pathological condition characterized by hypomagnesaemia and low plasma Ca concentrations (<1.4 mmol/L). Milk fever typically occurs around calving when there is a sudden increase in Ca losses through milk. Subclinical hypomagnesaemia reduces the ability of cows to mobilise calcium in response to hypocalcemia. In particular, Mg is required and involved in Ca absorption from the gut and Ca mobilization from bones, in order to maintain Ca homeostasis in plasma [4].

Apart from Mg deficient conditions, Mg supplementation is crucial to sustain ruminants’ performance. Mg requirement of modern dairy cows has increased, partly due to increased use of nitrogen (N) and potassium (K) fertilizers, and partly due to an increase in cow genetic merit. All cows are to some extent deficient in Mg in late pregnancy and early lactation. High producing cows (typically producing more than 40 kg of milk per day) are more at risk of Mg deficiency. 

Due to pasture and forage consumption by ruminants, Mg in soil is important in defining Mg availability for these animals. Mg content in soil differs between the various soil types and its availability to plants is influenced by several factors such as soil pH, organic matter content and fertilization [31]. This latter is an important feature on which depends the availability of minerals, including Mg. It has been observed that fertilization of soil with MgO provided and increased Mg content in grass, but it was considered insufficient to prevent Mg deficiency. Instead of this approach, direct Mg supplementation in cows’ diets is considered the best practice to prevent grass tetany and milk fever [5,8,9]. 

### 5.1. Dietary Interactions on Mg Absorption

**Figure 1:**
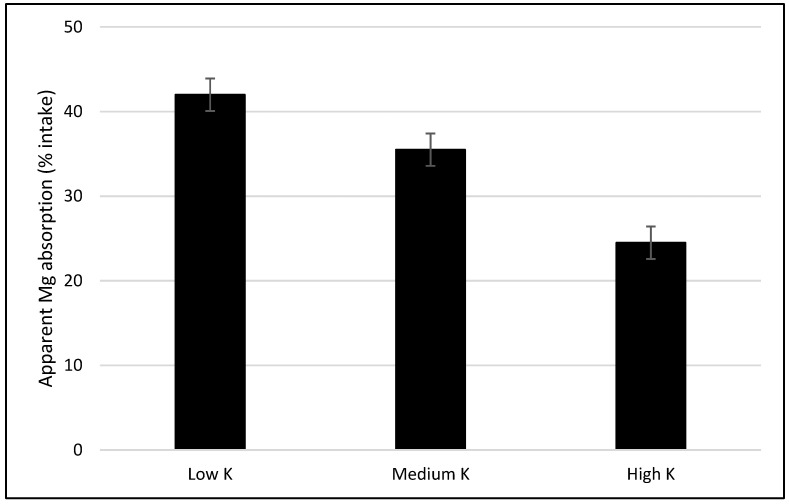
The effect of different dietary levels of K on apparent absorption of Mg (% intake) in sheep. Levels of K are expressed as g/kg dry matter (DM). Low K: 15.7 g/kg DM; medium K: 37.6 g/kg DM; high K: 77.4 g/kg DM. Standard error mean = 1.92. Data from [33].

There are some dietary interactions between single components of feedstuffs, such as minerals, and Mg absorption. One of the most known in ruminants is a negative interaction between K intake and Mg absorption at ruminal level, as seen by the use of manure as fertilizer. The rumen is an important site of Mg absorption for cows [4]. Indeed, at low K level in ruminal epithelial cells, the apical membrane potential provides a driving force for Mg uptake by the cells, whereas at high ruminal K level there is a depolarization of the membrane potential, thereby causing a reduction in Mg uptake by cells. It can be assumed that ruminal K concentration is linked to apical membrane potential [4,8,32]. This phenomenon was clearly observed in sheep, in which an increase of 1 g/kg DM in dietary K concentration decreased Mg absorption by 0.3% [33] (Figure 1). Mg absorption occurs also in small intestine at duodenal level, although a minor absorption rate is observed also in the large intestine.

Furthermore, Na deficiency is also linked to lowered Mg absorption, because Na level decreases at the expense of K level, thereby resembling the condition of high K level that impairs Mg absorption. Finally, it has been observed that starch supplementation increases Mg absorption in rats and humans [34]. This effect has not been observed in cows yet, but the reason could be that the intake of high amounts of carbohydrates, such as starch, could cause a decrease in ruminal pH, thereby raising Mg solubility and consequently its absorption.

### 5.2. Prevention of Mg Deficiency

The prevention of Mg deficiency must be performed both at short and long term, in order to prevent acute and chronic adverse conditions related to Mg deficiency. If there is a sudden need to avoid Mg deficiency, it is recommended to raise the dietary Mg content to adequate levels through the use of compound feeds. There are three main different forms of Mg that are used in ruminants’ compound feed: Mg sulphate, Mg chloride, and Mg oxide. Mg sulphate is considered a good bioavailable source of Mg as well as Mg oxide, which is the most common source of Mg used to prevent milk fever. Both Mg sulphate and Mg chloride can contribute to decreasing the so-called dietary cation-anion difference (DCAD), commonly calculated as ((Na^+^ + K^+^) − Cl^−^ + S^2−^) and expressed in milliequivalents (mEq). When Mg sulphate or Mg chloride are used as a source of supplemental Mg, their accompanying anions can reduce that balance, even if in terms of bioavailability Mg chloride should be intuitively preferred to both manipulate DCAD and prevent milk fever in dairy cows [8].

Mg supplementation in ruminants’ feeding is important both to sustain the metabolic activity of the enzymes that use Mg as cofactor and to prevent hypomagnesaemic clinical conditions such as grass tetany and milk fever. Mg intake and absorption in small intestine are strictly correlated and are subject to the influence of several factors, of which K level is one of the most important: a high K intake inhibits Mg absorption, thus increasing the risk of Mg deficiency. The K-induced inhibitory mechanism can be counteracted using supplemental dietary Mg to raise Mg level at short and long term.

## 6. Magnesium in Human Nutrition

### 6.1. Animal-Derived Food as Source of Dietary Mg

Mg supplementation in farm animals’ diets ensures an adequate Mg content in animal derived foods and consequently the Mg intake from these foods for humans. Whilst in the typical European diet cereals or cereal-derived foods are the largest source of Mg intake, animal-derived foods also make an important contribution. Typically, the recommended dietary intake of Mg for humans is around 300–400 mg/day. However, the reference values vary in relation to age and sex. For example, the recommended dietary intake for adult males is 350 mg/day, whereas for adult females is 300 mg/day [35]. Table 4 summarizes the contribution that animal-derived foods make to Mg intake in a selection of studies in several European countries. The data relate primarily to adults and some are relatively old but broadly indicate that meat, milk and dairy products make the largest contribution, with some notable differences between countries. The contributions seen in these studies contrast considerably with the values from the Mediterranean Healthy Eating, Ageing and Lifestyle (MEAL) study in Sicily which reported contributions of only 7, 4, 3 and 0% from milk and dairy products, fish, meat, and eggs, respectively [36]. In addition, the data in Table 4 mask the substantial variation in the supply of Mg that age of populations can make. For example, in the recent UK National Diet and Nutrition Survey (NDNS), milk and dairy products provide 25, 15, and 13% of Mg intake of children aged 1.5–3 and 4–10 years and subjects aged ≥75 years, respectively, compared with 9% in adults aged 19–64 years [37].

It is noteworthy that milk makes a greater contribution to Mg intake in very young and elderly subjects who are likely to be at greater risk of sub-optimal nutrition and will benefit from the high bioavailability of Mg in milk. A number of studies have shown that lactose in dairy products can enhance intestinal absorption of Mg in infants [41] and animal models [35]. This enhancement of Mg absorption has been attributed to the lowering of pH in the ileum by lactose fermentation which reduces the synthesis of insoluble Ca-Mg-phosphate complexes thus increasing absorption of Mg in the ileum. The benefits of lactose in this regard will of course be lost to subjects that are lactose intolerant and thus choose lactose-free dairy products. Table 5 summarizes the content of Mg in several animal-derived foods.

Whilst the data in Table 5 consistently show the importance of milk and meat as dietary sources of Mg, they do not reflect differences in Mg intake with some recent trends giving rise for concern. For example, in the recent UK NDNS, Roberts et al. [37] report that 50, 14, and 27% of adolescent females (11–18 years), adult females (19–64 years), and elderly females (≥75 years), respectively, have Mg intakes below the Lower Reference Nutrient Intake (LRNI). Equivalent values for males (27, 11, and 22%) are less extreme but are also concerning. The LRNI is that which is assumed to satisfy the nutrient requirements of the bottom 2.5% of the population so intakes considerably lower than this reflect how serious this situation is. It is noteworthy that in the UK milk and red meat consumption, especially by young females, has reduced over recent decades and this will have contributed to the substantially suboptimal intake of Mg and some other nutrients currently seen [45]. It is also interesting that the European Food Safety Authority (EFSA) [13] has recommended what it describes as ‘adequate intakes’ of Mg which for children aged 3 to 15 years are substantially higher than the UK Reference Nutrient Intakes for that age group.

The role of Mg as a cofactor in many body enzyme systems has been known for some time. Many of these involve adenosine triphosphate (ATP), which is involved in a wide range of biochemical pathways including intermediary metabolism related to the synthetic pathways for carbohydrates, lipids and proteins. About 60% of body Mg is in bone [46] and some 25% is in muscle mitochondria [47] and it is now becoming clear that its role in the musculoskeletal system is vital in relation to diet-related chronic diseases [48].

### 6.2. Mg and Bone Health

Whilst it has been recognised for some considerable time that adequate intakes of protein and Ca together with an optimum vitamin D status are important prerequisites for bone development it is now becoming clear that Mg also has a crucial role. Research with children aged 4–8 years reported that Ca intake, when not very sub-optimal, was not substantially linked to bone mineral status, whereas Mg intake, and particularly the amount absorbed, were important predictors of bone mineral density and bone mineral content [49]. The authors highlight that this work provides good evidence that Mg should be more considered as an important nutrient in relation to bone development. In addition, more recently the Japanese Kuopio Ischemic Heart Disease prospective study has shown that low serum Mg concentrations in men aged 42–61 years were associated with increased bone fracture risk [50]. To what extent these findings are relevant to other populations is uncertain at present, but ensuring that adequate Mg intake is clearly and especially important during the phase of rapid bone growth in late childhood/ and early adolescence. Mg is now also known to have a considerable interaction with vitamin D being an essential cofactor for vitamin D synthesis and its subsequent activation, which in turn can increase intestinal absorption of Mg [51]. This further highlights the importance of Mg in bone health. Given the co-existence of sub-optimal vitamin D status, the substantially sub-optimal Mg intakes in UK female adolescents noted above is a matter of substantial concern.

There is also increasing evidence of a benefit of Mg for bone health in later life. Erem et al. [52] reviewed studies which showed that the risk of osteoporosis in older subjects can be a consequence of low Mg intake. This can lead to excess Ca release from the bones with the resultant increased excretion leading to increased bone fragility and hence a higher risk of bone fractures. In addition, high intakes of Ca can lead to lower retention of Mg and it has been proposed that the optimal dietary ratio of Ca:Mg is between 2.0:1.0 and 2.8:1.0 [52] but they highlight that in a lot of current US diets the ratio above 3.0:1.0. 

There is clearly an urgent need for further research on the interaction of Mg with Ca and vitamin D in relation to bone development in the young and bone strength in the elderly. It is well known that milk and dairy products are excellent sources of Ca and, as noted above, also an important source of Mg for the young and elderly, as well as being an excellent vehicle for vitamin D fortification.

### 6.3. Mg and Sarcopenia

Sarcopenia is a condition mainly associated with chronic loss of muscle mass and muscle function with advancing age [53]. It also predicts functional decline, hospitalization, and living in community dwelling for the elderly. It is therefore a condition of increasing importance in the elderly (although it can occur in middle age) with an increasing prevalence associated with the increasing age of many populations worldwide. The condition can have consequences additional to simple muscle loss, as for example, it reduces the protection of the bone with increased risk of bone breakage in a fall which can have an immense effect on mobility, disability and general quality of life. A less well appreciated outcome of reduced muscle mass and the associated reduced mobility is the increased risk of metabolic diseases, particularly type 2 diabetes [54]. Since skeletal muscles are the major site of glucose uptake and clearance from the circulation, reduction in muscle mass can adversely affect glycemic control [55].

As with the influence of Mg intake on bone mineralization noted earlier, there is also increasing evidence of an association between Mg and preservation and functionality of skeletal muscle. Dominguez et al. [56] used baseline data from the prospective study named “Invecchiare in Chianti” (InCHIANTI, Aging in the Chianti area of Tuscany) on risk factors for late-life disability. They selected 1138 men and women (aged 66.7 ± 15.2 y) with full data on muscle performance and blood Mg. After adjustments for key confounders (age, sex, etc.) serum Mg concentrations were significantly and positively associated with muscle performance as assessed by measures including grip strength (*p* = 0.0002), lower leg muscle power (*p* = 0.001), and knee extension torque (*p* < 0.0001). More recently Welch et al. [57] studied the cross-sectional associations between Mg intake and skeletal muscle mass (expressed as fat-free mass (FFM) as a percentage of body weight (FFM%)) and grip strength in 56,575 males and females aged 39–72 years from the UK Biobank cohort. They found positive associations between quintiles of Mg intake and grip strength (*p* trend < 0.001) and FFM% (*p* trend < 0.001). They reported that the relationship with grip strength was stronger for men ≥60 years of age than in younger men, although the opposite was the case for women. The authors indicated that this study was the largest population to date used to study the association between Mg intake and direct functionality measures of skeletal muscle. 

Zhang et al. [58] reviewed the evidence from animal and human studies as to whether Mg can enhance performance during exercise. They concluded that animal studies showed that Mg might improve exercise performance, possibly by increasing glucose availability to the brain and muscles whilst lowering and delaying lactate accumulation in the muscles. They found that human studies had primarily examined physiological effects such as blood pressure, heart rate and maximal oxygen uptake (VO_2_ max) rather than direct muscle performance but they did report evidence that Mg supplementation might enhance some performance parameters in both aerobic and anaerobic exercise regimes. Despite blood only containing about 1% of total body Mg, serum Mg concentration has been used as a measure of Mg status in most studies. Recently however, Cameron et al. [59] showed that the measurement of intramuscular ionised Mg using phosphorus magnetic resonance spectroscopy (^31^PMRS) was positively associated with knee-extension strength (*p* < 0.001 in women; *p* = 0.003 in men), while total serum Mg was not associated with muscle strength (*p* = 0.27). The authors propose that intramuscular ionised Mg by ^31^PMRS is a superior measure of Mg status than total serum Mg, perhaps particularly when muscle weakness of an uncertain cause is found.

Clearly more work on the increasingly important relationship between Mg and muscle function is needed. Given the substantially sub-optimal Mg intakes in elderly populations such as in the UK [37] and the US [52], and the increasing prevalence of sarcopenia, this work is now urgent.

### 6.4. Mg and Cancer Risk

**Figure 2:**
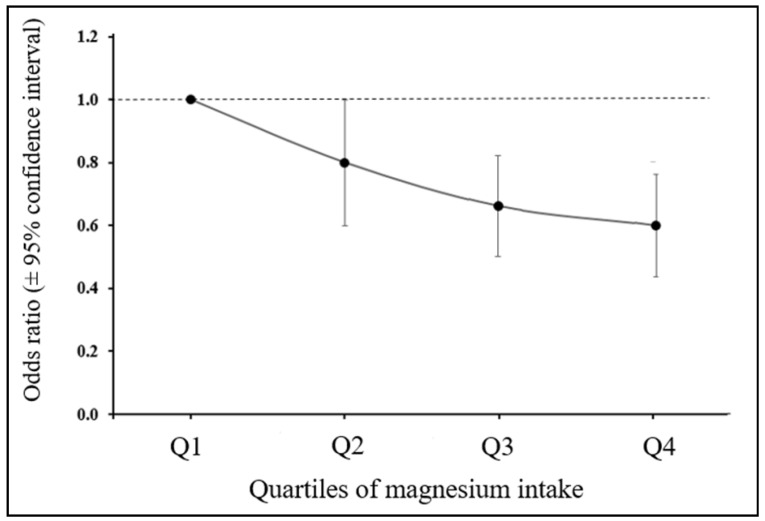
Dose-response association between Mg intake and risk of breast cancer in Chinese women. Derived from [60].

Although this area of work is relatively new there is an increasing interest in the possible association between Mg status and cancer risk. The recent case-control study of Huang et al. [60] explored the effect of dietary Mg intake on breast cancer risk directly and indirectly via the effect of Mg on the inflammatory markers C-reactive protein (CRP) and interleukin-6 (IL-6). Multivariable logistic regression was used to assess the odds ratio (OR) and 95% confidence interval (95% CI), together with path analysis to explore mediating effects. The results showed that a higher Mg intake (≥280 mg/d) was associated with a significantly lower risk of breast cancer (OR 0.80, 95% CI 0.65, 0.99) than intakes <280 mg/day and there was an overall dose-response between Mg intake and breast cancer risk (Figure 2). Additionally, circulating CRP concentration was positively associated with the risk of breast cancer (OR 1.43, 95% CI 1.02, 2.01). IL-6 showed no association with breast cancer risk but the path analysis identified that dietary Mg influenced breast cancer risk directly and indirectly by its lowering effect on CRP. As the authors noted, this study was the first of its kind but had weaknesses including the well-recognised limitations of case-control studies plus the fact that the measurement of the inflammatory markers was only made in relatively small number of subjects (322 cases and controls). Nevertheless, this study clearly supports the objective of increasing Mg intake including some populations noted earlier with substantial sub-optimal Mg intakes. 

There is increasing evidence of an inverse association between vitamin D status (circulating 25(OH)D3) and mortality in colo-rectal cancer (CRC) patients and the meta-analysis of Maalmi et al. [61] involving 11 studies and 7718 CRC patients showed that those with the highest vitamin D status had significantly lower risk of all-cause mortality with a hazard ratio (HR) of 0.68 (95% CI: 0.55, 0.85) and CRC cause mortality (HR 0.67, 95% CI 0.57, 0.78) than those with the lowest vitamin D status. As noted earlier, Mg is heavily involved in biochemical pathways for vitamin D synthesis and the conversion of 25(OH)D3 to the active 1,25(OH)_2_D3 form of vitamin D. The study of Wesselink et al. [62] with 1169 newly diagnosed patients examined the associations between circulating 25(OH)D3 concentrations, Mg or Ca dietary intake (including supplements) and recurrence rate and all-cause mortality. Overall, the study concluded that having an adequate vitamin D status together with an adequate Mg intake is essential for reducing the risk of mortality in CRC patients although the wide applicability and exact mechanisms are not known and should be investigated. 

## 7. Conclusions 

Mg is required in animal nutrition because of its major role in cellular metabolism and bone development and further to avoid adverse health conditions that impair animals’ health and consequently their productivity. Usually, Mg minimum requirements are met only using common feed ingredients. However, the dramatic increase in productivity of high producing farm animals over the past decades has led to new challenges in nutritional requirements to support higher animal performance. For this reason, Mg supplementation in animal nutrition above the minimum requirements has been regarded as a best practice to face with higher performance, mainly in terms of fertility and product quality. Mg supplementation is essential also because it ensures an adequate Mg content in animal-source food. To summarize, Mg supplementation exerts beneficial effects in high producing farm animals in terms of productive and reproductive performances and is essential for their health and wellbeing. 

In human nutrition Mg is also essential. It is a cofactor in more than 300 enzyme systems which regulate diverse biochemical reactions in the body, including protein synthesis, muscle and nerve transmission, neuromuscular conduction, signal transduction, blood glucose control, and blood pressure regulation. In light of this, the impact of sub-optimal Mg intake by humans can be substantial as there is increasing evidence of its key role in bone development, muscle function and an association with some health risk. In this respect dietary intake and source become also important. It is clear that for many populations the animal-derived foods, and notably meat, milk and dairy products are important dietary sources of Mg [35]. This also seems to be particularly important in age groups which have substantial nutrient insecurity such as adolescents and the elderly. It is also becoming increasingly clear that Mg and vitamin D have an interdependence and are involved in the aetiology of several chronic diseases which have an increasing prevalence. Whilst much needs to be known about the association of Mg with risk of chronic diseases, a concerted effort should be made by public health bodies to ensure Mg intake and vitamin D status are satisfactory. 

Overall, the recommendation for both animals and humans is the same, do what is necessary to ensure an adequate dietary supply of Mg.

## Figures and Tables

**Table 1:** Production efficiency trend: feed conversion rate (FCR), kg feed per kg of animal product. Adapted from [11,12].

Product	1960–1970 FCR	Today FCR	Efficiency Improvement
Poultry meat	4.5	1.9	57%
Turkey meat	6.0	2.5	58%
Eggs	4.3	2.1	51%
Milk	2.2	0.7	68%
Pig (100 kg) meat	4.3	2.7	37%
Beef (400–700 kg)	9	7	22%
Mean	5.05	2.81	49%

**Table 2:** Mg content of mineral supplements. Adapted from [15].

Mg Source	Mg Content (g/100 g)
Mg oxide	50.5–52.0
Mg hydroxide	36.0–38.0
Mg phosphate	24.0–33.0
Mg chloride	12.0
Mg sulphate	10.0

**Table 3:** Recommended quantities of Mg (expressed per kg of metabolic body weight) in selected species.

Species	Body Weight (BW)	Mg of Mg/kg of Metabolic BW *	% (Relative to Humans)
Human (adult)	70 kg	12.4	100
Pig	100 kg	33.5	270.1
Poultry	3.5 kg	19.6	158
Cow	600 kg	0.25	2

* Metabolic BW = BW^0.75^.

**Table 4:** Contribution of animal-derived foods to Mg intake by adults.

Country	Study	Gender	Contribution to Mg Intake (%):				Reference
			Milk and Dairy Products	Meat and Products	Eggs	Fish and Products	
Italy	Total-diet ^1^	Mixed	11	13	NG ^2^	5	[38]
Italy	INRAN-SCAI, 2005–06	Mixed	12	10	1	5	[39]
Italy	EPIC	Men	6.8	10.0	0.1	2.4	[40]
Italy	EPIC	Women	9.0	9.3	0.2	2.3	[40]
United Kingdom	EPIC	Men	13.2	9.2	0.2	2.7	[40]
United Kingdom	EPIC	Women	14.1	7.9	0.2	2.7	[40]
United Kingdom	NDNS	Mixed 19–64 years	9	15	1	3	[37]
Greece	EPIC	Men	8.4	6.1	0.1	5.0	[40]
Germany	EPIC	Men	6.2	12.1	0.1	1.5	[40]
The Netherlands	EPIC	Men	10.2	11.8	0.2	1.2	[40]

^1^ Based on food purchases so will include children ^2^ No value given.

**Table 5:** Distribution of Mg content (mg/kg of fresh wt) in selected foods of animal origin. Adapted from [35,42,43,44].

Animal-Derived Food	Mg	Animal-Derived Food	Mg
Chicken (range)	140–210	Cow’s Milk (range)	86–100
Breast	210	Whole milk (3.25% fat)	98–110
Drumstick	196	Reduced Fat milk (2% fat)	98–111
Chicken meat products	135–142	Low fat milk (1% fat)	98–112
Pork (range)	195–290	Skim milk	98–113
Loin	207	Goat milk	139
Neck	212	Sheep milk	180
Hind leg	237	Dairy products (range)	20–425
Shoulder	195	Cream	60
Sausage	117–289	Butter	20
		Cheese	130–425

## Data Availability

Data sharing is not applicable to this article.
